# Supplementary material for: The Evolution and Characterization of the RNA Interference Pathways in Lophotrochozoa
Source: Genome Biol Evol. 2024 May 7;16(5):evae098. doi: 10.1093/gbe/evae098 (PMC11114477; doi:10.1093/gbe/evae098)
Supplement: evae098_Supplementary_Data [file evae098_supplementary_data.zip › Supplementary_materials.zip!/selected_miRNAs/NoGe_selected_miRNAs/mirdeep_pdf_files/NMRB01002279.1_Notospermus_geniculatus_strain_Ushimado_scaffold2279,_whole_genome_shotgun_sequence_37108.pdf]

Provisional ID	:	NMRB01002279.1_Notospermus_geniculatus_strain_Ushimado_scaffold2279,_whole_genome_shotgun_sequence_37108
Score total	:	43184.5
Score for star read(s)	:	3.9
Score for read counts	:	43176.2
Score for mfe	:	1.4
Score for randfold	:	0
Score for cons. seed	:	3
Total read count	:	84700
Mature read count	:	84672
Loop read count	:	0
Star read count	:	28

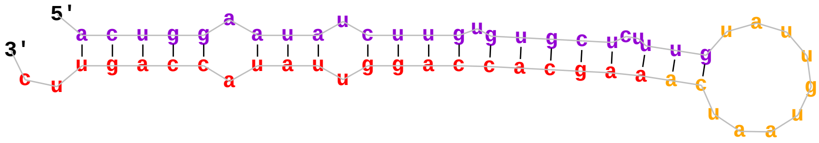

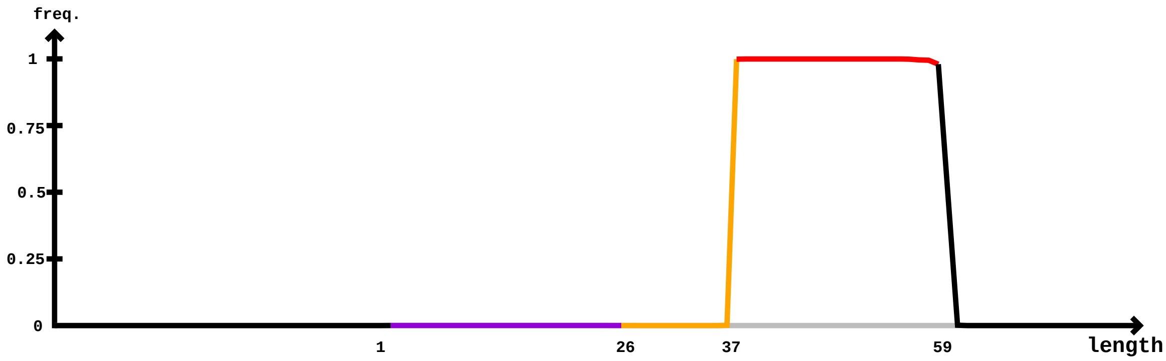


```
                                   Star                                 Mature
5'- aaggcuuacccuguccuagcaucaguuuugcgaacuggaauaucuugugugcucuuuguauuguaaucaaagcaccagguuauaccaguucgugaucugguccaugcaaua   -3'    obs
    aaggcuuacccuguccuagcaucaguuuugcgaacuggaauaucuugugugcucuuuguauuguaaucaaagcaccagguuauaccaguucgugaucugguccaugcaaua          exp
    .((((.......).))).(((((((..(((((((((((.(((.((((.(((((..(((.........)))))))))))).))).))))))))))).)))....))))....   reads    mm    sample
    ............Auccuagcaucaguuuugcga..............................................................................   3        1     Fm3
    .................................acuggaauaucuugugugcucuu.......................................................   1        0     Fm3
    ....................................................................Caagcaccagguuauaccaguuc....................   1        1     Fm3
    .....................................................................aagcaccagguuauacca........................   19       0     Fm3
    .....................................................................aGgcaccagguuauaccag.......................   1        1     Fm3
    .....................................................................aagcaccGgguuauaccag.......................   1        1     Fm3
    .....................................................................aagcaccagguuauaccaU.......................   5        1     Fm3
    .....................................................................aagcaccagguuauaccCg.......................   2        1     Fm3
    .....................................................................aagcaccaUguuauaccag.......................   1        1     Fm3
    .....................................................................aagcaccagguuauaccag.......................   195      0     Fm3
    .....................................................................aagcaccagguuauacAag.......................   2        1     Fm3
    .....................................................................aagcaccagguuauaccUgu......................   1        1     Fm3
    .....................................................................aagcaccagguuauaccagC......................   1        1     Fm3
    .....................................................................Uagcaccagguuauaccagu......................   1        1     Fm3
    .....................................................................aagcaccagguuauaccagu......................   27       0     Fm3
    .....................................................................aagcaccagguuauaUcagu......................   3        1     Fm3
    .....................................................................aagcaccagguuauacGaguu.....................   1        1     Fm3
    .....................................................................aagcaccagguGauaccaguu.....................   2        1     Fm3
    .....................................................................aagcaccagguuauaccaguu.....................   334      0     Fm3
    .....................................................................aagcaccaggAuauaccaguu.....................   2        1     Fm3
    .....................................................................Gagcaccagguuauaccaguu.....................   1        1     Fm3
    .....................................................................aagcaccagguuauacUaguu.....................   2        1     Fm3
    .....................................................................aagcaccagguuauaccGguu.....................   2        1     Fm3
    .....................................................................aagcaccagguuauacAaguu.....................   2        1     Fm3
    .....................................................................aagAaccagguuauaccaguu.....................   1        1     Fm3
    .....................................................................aagcaccagguuauaccUguu.....................   2        1     Fm3
    .....................................................................aagcaccagguuauGccaguu.....................   1        1     Fm3
    .....................................................................aagcaAcagguuauaccaguu.....................   1        1     Fm3
    .....................................................................aagcaccaggGuauaccaguu.....................   1        1     Fm3
    .....................................................................aagcaccagguuaCaccaguuc....................   1        1     Fm3
    .....................................................................aagcaccagguuauaccagAuc....................   2        1     Fm3
    .....................................................................aagcaccagguuaAaccaguuc....................   1        1     Fm3
    .....................................................................aagAaccagguuauaccaguuc....................   2        1     Fm3
```

Star Mature

aaggcuuacccuguccuagcaucaguuuugcgaacuggaauaucuugugugcucuuuguauuguaaucaaagcaccagguuauaccaguucgugaucugguccaugcaaua			
....aagcaccagguuauaccaguuU....................	5	1	Fm3
....aagcaccagguuauaccaguAc....................	1	1	Fm3
....aagcacAagguuauaccaguuc....................	2	1	Fm3
....aagGaccagguuauaccaguuc....................	2	1	Fm3
....aagcaccagguuauaccGguuc....................	10	1	Fm3
....aagcaccagguuauaccUguuc....................	12	1	Fm3
....aagcaccaggAuauaccaguuc....................	9	1	Fm3
....aaCcaccagguuauaccaguuc....................	1	1	Fm3
....aagcaccagguuauaccaguGc....................	1	1	Fm3
....aaAcaccagguuauaccaguuc....................	1	1	Fm3
....aagcaccagguuauacUaguuc....................	10	1	Fm3
....aagUaccagguuauaccaguuc....................	1	1	Fm3
....aagcaccagguuauaccCguuc....................	4	1	Fm3
....aaUcaccagguuauaccaguuc....................	1	1	Fm3
....aagcaccaggGuauaccaguuc....................	3	1	Fm3
....aagcaccagguCauaccaguuc....................	2	1	Fm3
....aagcaccagguuauaccaguuc....................	2489	0	Fm3
....aagcaccagguuauacGaguuc....................	2	1	Fm3
....aagcaccagguuauaUcaguuc....................	1	1	Fm3
....Gagcaccagguuauaccaguuc....................	1	1	Fm3
....aagcaccGgguuauaccaguuc....................	1	1	Fm3
....aagcaccagguuauaccaguuG....................	1	1	Fm3
....aCgcaccagguuauaccaguuc....................	1	1	Fm3
....aagcaccagguuauacAaguuc....................	12	1	Fm3
....aagcaccagguuGuaccaguuc....................	1	1	Fm3
....aagcaccaggCuauaccaguuc....................	2	1	Fm3
....aagcaccagguuauaccaguucC...................	3	1	Fm3
....aagcaccagguuauaccaguucU...................	74	1	Fm3
....aagcaccagguuauaccaguucUu..................	1	1	Fm3
....agcaccagguuauaccaguuc....................	1	0	Fm3
.................................acuggaauaucuugugugcucuu...	1	0	Mm3
.................................acuggaauaucuugugugcucuuug...	6	0	Mm3
....uauuguaaucaaagcaccagguuauacc.........................	1	0	Mm3
....aaagcaccagguuauaccaguu.....................	2	0	Mm3
....aaagcaccagguuauaccaguuc....................	1	0	Mm3
....Caagcaccagguuauaccaguuc....................	2	1	Mm3
....aagcaccagguuauacca........................	5	0	Mm3
....aagcaccagguuauaccagu......................	10	0	Mm3
....aagcaccagguuauaccaAuu.....................	1	1	Mm3
....aagcaccagguuauaccaguu.....................	104	0	Mm3
....aagcaccagguuauacAaguu.....................	1	1	Mm3
....aagcaccGgguuauaccaguuc....................	2	1	Mm3
....Cagcaccagguuauaccaguuc....................	1	1	Mm3
....aagUaccagguuauaccaguuc....................	1	1	Mm3
....aagcaUcagguuauaccaguuc....................	1	1	Mm3
....aagcaccagguuauaccaguuA....................	3	1	Mm3
....aagcaccagguuauaccaUuuc....................	1	1	Mm3
....aagcaccagguuauacGaguuc....................	13	1	Mm3
....aagcaccaggGuauaccaguuc....................	6	1	Mm3
....aagcaccagguuauaccGguuc....................	21	1	Mm3
....aagAaccagguuauaccaguuc....................	1	1	Mm3
....aagcaccagguuauUccaguuc....................	2	1	Mm3
....aagcaccagguuauaccaguU....................	9	1	Mm3
....aagcaccaUguuauaccaguuc....................	1	1	Mm3
....aagcaccagUuuauaccaguuc....................	2	1	Mm3
....aagcaccagguuauGccaguuc....................	2	1	Mm3
....aagcacUagguuauaccaguuc....................	3	1	Mm3
....Nagcaccagguuauaccaguuc....................	1	1	Mm3
....aaAcaccagguuauaccaguuc....................	1	1	Mm3
....aagcaccagguuauaccagCuc....................	1	1	Mm3
....aUgcaccagguuauaccaguuc....................	3	1	Mm3
....aaUcaccagguuauaccaguuc....................	1	1	Mm3
....aagcaccagguuauaccUguuc....................	9	1	Mm3
....aagcaccagguuauaUcaguuc....................	1	1	Mm3
....aagcaNcagguuauaccaguuc....................	2	1	Mm3
....Uagcaccagguuauaccaguuc....................	4	1	Mm3
....aagcaccagguuauaccaguCc....................	1	1	Mm3
....aagcaccagguuauaccaguuc....................	5641	0	Mm3
....aagcaccaggCuauaccaguuc....................	7	1	Mm3

Star / Mature

aaggcuuacccuguccuagcaucaguuuugcgaacuggaauaucuugugugcucuuuguauuguaaucaaagcaccagguuauaccaguucgugaucugguccaugcaaua			
....aagcaccagguuauacUaguuc....................	30	1	Mm3
....Gagcaccagguuauaccaguuc....................	2	1	Mm3
....aagcaccagguuaAaccaguuc....................	1	1	Mm3
....aagcaccagguuaCaccaguuc....................	2	1	Mm3
....aagcaccaggAuauaccaguuc....................	14	1	Mm3
....aagcaccUgguuauaccaguuc....................	2	1	Mm3
....aagcaccaAguuauaccaguuc....................	1	1	Mm3
....aagcaccagguuauacAaguuc....................	21	1	Mm3
....aagcaccagguuauaccCguuc....................	24	1	Mm3
....aagcaccagguuauaccaguucU...................	290	1	Mm3
....aagcaccagguuauaccaguucC...................	4	1	Mm3
....aagcaccagguuauaccaguucCu..................	1	1	Mm3
....aagcaccagguuauaccaguucUu..................	7	1	Mm3
....agcaccagguuauaccaguuc....................	7	0	Mm3
....gcaccagguuauaccaguuc....................	1	0	Mm3
.................................acuggaauUucuugugugcucuuug...	1	1	Fm1
....aaagcaccagguuauaccaguuc....................	6	0	Fm1
....Caagcaccagguuauaccaguuc....................	6	1	Fm1
....aagcaccagguuauacca........................	13	0	Fm1
....aagcaccagguuauaccag.......................	2	0	Fm1
....aagcaccagguuauaccagu......................	13	0	Fm1
....aagcaccagguuauaccaguu.....................	92	0	Fm1
....aagcaccagguuauacAaguu.....................	2	1	Fm1
....aagcaccagguuauaccGguu.....................	1	1	Fm1
....aagcaccagguuauaccUguu.....................	1	1	Fm1
....aagcaNcagguuauaccaguu.....................	1	1	Fm1
....aagcaccagUuuauaccaguuc....................	1	1	Fm1
....aagUaccagguuauaccaguuc....................	3	1	Fm1
....aagcaccagguuauaccaguuU....................	12	1	Fm1
....aagcaccagguuauaUcaguuc....................	4	1	Fm1
....aagcaccagguuauaccaguuA....................	10	1	Fm1
....aagcaccaggAuauaccaguuc....................	31	1	Fm1
....aagcaccagguuauaccaguuc....................	13386	0	Fm1
....aagcaccagguuauGccaguuc....................	6	1	Fm1
....Gagcaccagguuauaccaguuc....................	5	1	Fm1
....Uagcaccagguuauaccaguuc....................	9	1	Fm1
....aagcaUcagguuauaccaguuc....................	4	1	Fm1
....aagcaccaUguuauaccaguuc....................	2	1	Fm1
....aagcaccagguuauacGaguuc....................	25	1	Fm1
....aagcaccagguuUuaccaguuc....................	1	1	Fm1
....aagcaccagguuauacUaguuc....................	32	1	Fm1
....aagGaccagguuauaccaguuc....................	1	1	Fm1
....aagcaccGgguuauaccaguuc....................	17	1	Fm1
....aagcaccagguuauaccaguGc....................	1	1	Fm1
....aagcaccagguuaGaccaguuc....................	1	1	Fm1
....aagcaccaggCuauaccaguuc....................	16	1	Fm1
....aagcaccagguuauacAaguuc....................	62	1	Fm1
....aagcaccUgguuauaccaguuc....................	1	1	Fm1
....aagcUccagguuauaccaguuc....................	2	1	Fm1
....aagcaccaggGuauaccaguuc....................	19	1	Fm1
....aagcaccagguuauUccaguuc....................	1	1	Fm1
....aagcaccagguuauaccaUuuc....................	3	1	Fm1
....aagcacUagguuauaccaguuc....................	5	1	Fm1
....aUgcaccagguuauaccaguuc....................	3	1	Fm1
....aaAcaccagguuauaccaguuc....................	3	1	Fm1
....aagcaNcagguuauaccaguuc....................	4	1	Fm1
....aagcaccagguGauaccaguuc....................	2	1	Fm1
....aagcaccagguuauaccUguuc....................	37	1	Fm1
....aagcacAagguuauaccaguuc....................	3	1	Fm1
....aagcaccagguuauaccCguuc....................	33	1	Fm1
....aaUcaccagguuauaccaguuc....................	3	1	Fm1
....aagAaccagguuauaccaguuc....................	8	1	Fm1
....aagcaccagguuauaccGguuc....................	54	1	Fm1
....aagcaccaCguuauaccaguuc....................	1	1	Fm1
....aCgcaccagguuauaccaguuc....................	1	1	Fm1
....aagcGccagguuauaccaguuc....................	3	1	Fm1
....aGgcaccagguuauaccaguuc....................	2	1	Fm1
....aagcCccagguuauaccaguuc....................	2	1	Fm1
....aagcaccagguCauaccaguuc....................	2	1	Fm1

Star **Mature**

aaggcuuacccuguccuagcaucaguuuugcgaacuggaauaucuugugugcucuuuguauuguaaucaaagcaccagguuauaccaguucgugaucugguccaugcaaua			
....Nagcaccagguuauaccaguuc....................	5	1	Fm1
....aagcaccagguuGuaccaguuc....................	1	1	Fm1
....aaCcaccagguuauaccaguuc....................	6	1	Fm1
....aagcaccagguuauaccaguAc....................	1	1	Fm1
....aagcaccagguuauaccaguucg...................	9	0	Fm1
....aagcaccagguuauaccaguucU...................	745	1	Fm1
....aagcaccagguuauaccUguucg...................	1	1	Fm1
....aagcaccagguuauaccaguucC...................	85	1	Fm1
....aagcaccagguuauaccaguucUu..................	49	1	Fm1
....aagcaccagguuauaccaguucCu..................	5	1	Fm1
....agcaccagguuauaccaguuc....................	2	0	Fm1
.................................acuggaauaucuugugugcucu..	6	0	Fm2
.................................acuggaauaucuugugugcucuuug...	1	0	Fm2
....aaagcaccagguuauaccaguuc....................	8	0	Fm2
....Caagcaccagguuauaccaguuc....................	2	1	Fm2
....aagcaccagguuauaccagu......................	4	0	Fm2
....aagcaccagguuauaccaUu......................	2	1	Fm2
....aagcaccagguuauaccaguu.....................	78	0	Fm2
....aagcaccagguuauaccCguu.....................	1	1	Fm2
....aagcaccagguuaAaccaguu.....................	1	1	Fm2
....aagcaccagguuauaccaUuuc....................	1	1	Fm2
....aagcGccagguuauaccaguuc....................	1	1	Fm2
....aagcaccagguuauaccaguuc....................	8938	0	Fm2
....aagcaccagguuaCaccaguuc....................	2	1	Fm2
....aagcaccagguuauUccaguuc....................	1	1	Fm2
....aagcaccaggCuauaccaguuc....................	16	1	Fm2
....aagcaccagguuauaccCguuc....................	30	1	Fm2
....aagcaccagguuauaccUguuc....................	22	1	Fm2
....aagcaccagguuauacAaguuc....................	31	1	Fm2
....aagcaccaCguuauaccaguuc....................	1	1	Fm2
....Gagcaccagguuauaccaguuc....................	2	1	Fm2
....aagcaccagguuauacUaguuc....................	28	1	Fm2
....aagcaccagguuauaccaguuA....................	11	1	Fm2
....aagcacUagguuauaccaguuc....................	2	1	Fm2
....aagcaNcagguuauaccaguuc....................	1	1	Fm2
....aagcaccagguuauGccaguuc....................	1	1	Fm2
....aagcaccagguuauaccagCuc....................	2	1	Fm2
....aagcaccagUuuauaccaguuc....................	1	1	Fm2
....aagcaccagguuauaccGguuc....................	34	1	Fm2
....aagcaccaggAuauaccaguuc....................	19	1	Fm2
....aagcacAagguuauaccaguuc....................	1	1	Fm2
....aUgcaccagguuauaccaguuc....................	1	1	Fm2
....aagcaccagguuGuaccaguuc....................	1	1	Fm2
....aagcaccagguuauaccaguuG....................	1	1	Fm2
....Nagcaccagguuauaccaguuc....................	6	1	Fm2
....Uagcaccagguuauaccaguuc....................	5	1	Fm2
....aagcaccagguuauacGaguuc....................	9	1	Fm2
....aagAccagguuauaccaguuc....................	1	1	Fm2
....aGgcaccagguuauaccaguuc....................	2	1	Fm2
....aagcaccagguGauaccaguuc....................	1	1	Fm2
....aagcaccagguuauaccaguGc....................	1	1	Fm2
....aagcaccaggGuauaccaguuc....................	6	1	Fm2
....aagcaccagguuauaUcaguuc....................	1	1	Fm2
....aagcaccCgguuauaccaguuc....................	1	1	Fm2
....aagcCccagguuauaccaguuc....................	1	1	Fm2
....aagcaccagguuUuaccaguuc....................	1	1	Fm2
....aagcaccGgguuauaccaguuc....................	6	1	Fm2
....aagcaccagguuauaccagAuc....................	2	1	Fm2
....aagcaccagguuaAaccaguuc....................	1	1	Fm2
....aagcaUcagguuauaccaguuc....................	1	1	Fm2
....aagcacNagguuauaccaguuc....................	1	1	Fm2
....aagcaccagguuauaccaguucC...................	35	1	Fm2
....aagcaccagguuauaccaguucU...................	491	1	Fm2
....Nagcaccagguuauaccaguucg...................	1	1	Fm2
....aagcaccagguuauaccaguucUu..................	11	1	Fm2
....agcaccagguuauaccaguuc....................	3	0	Fm2
....agcaccagguuauaccaguucU...................	5	1	Fm2
....caccagguuauaccaguuc....................	3	0	Fm2
.................................acuggaauaucuugugugcucuu...	2	0	Mm2

	Star		Mature			

aaggcuuacccuguccuagcaucaguuuugcgaacuggaauaucuugugugcucuuuguauuguaaucaaagcaccagguuauaccaguucgugaucugguccaugcaaua			
.................................acuggaauaucuugugugcucuuu..	4	0	Mm2
.................................acuggaauaucuugugugcucuuug...	6	0	Mm2
....uauuguaaucaaagcaccagguu..............................	2	0	Mm2
....aaagcaccagguuauaccaguuc....................	13	0	Mm2
....Caagcaccagguuauaccaguuc....................	23	1	Mm2
....aagcaccagguuauacca........................	11	0	Mm2
....aagcaccagguuauaccag.......................	10	0	Mm2
....aagcaccagguuauaccUg.......................	1	1	Mm2
....aagcaccagguuauaccaU.......................	1	1	Mm2
....aagcaccagguuauaccUgu......................	1	1	Mm2
....aagcaccagguuauaccagu......................	36	0	Mm2
....aagcaccagguuauaccCguu.....................	2	1	Mm2
....aagcaccagguuauaccGguu.....................	1	1	Mm2
....aagcaccagguuauaccUguu.....................	1	1	Mm2
....aagcaccagAuuauaccaguu.....................	1	1	Mm2
....aagcaccagguuauacUaguu.....................	1	1	Mm2
....aagcaccaggAuauaccaguu.....................	1	1	Mm2
....aagcaccaggGuauaccaguu.....................	1	1	Mm2
....aagcaccagguuauaccaguu.....................	346	0	Mm2
....aagcaccagguuauacAaguu.....................	4	1	Mm2
....aagcaccGgguuauaccaguuc....................	23	1	Mm2
....Uagcaccagguuauaccaguuc....................	21	1	Mm2
....aUgcaccagguuauaccaguuc....................	9	1	Mm2
....aagcaNcagguuauaccaguuc....................	5	1	Mm2
....aagcaccagguuauacUaguuc....................	101	1	Mm2
....aagcaccagguuauaccagCuc....................	3	1	Mm2
....aagcaccaCguuauaccaguuc....................	1	1	Mm2
....aagcaccagguuauaccGguuc....................	111	1	Mm2
....aagcaccagguuaCaccaguuc....................	8	1	Mm2
....aagcaccagguuauaccaguAc....................	1	1	Mm2
....aagcaccagguuauaccaguCc....................	5	1	Mm2
....aagcaccagguGauaccaguuc....................	3	1	Mm2
....aagcaccagguuauaccaguuc....................	39840	0	Mm2
....aagcaccagguuauaccaAuuc....................	3	1	Mm2
....aagcaccagguuauUccaguuc....................	7	1	Mm2
....aagcCccagguuauaccaguuc....................	3	1	Mm2
....aaUcaccagguuauaccaguuc....................	9	1	Mm2
....aagAaccagguuauaccaguuc....................	10	1	Mm2
....Nagcaccagguuauaccaguuc....................	21	1	Mm2
....aagcaccagguuUuaccaguuc....................	6	1	Mm2
....aagcaccagguuauaccagAuc....................	5	1	Mm2
....aagcaccaggAuauaccaguuc....................	77	1	Mm2
....aagGaccagguuauaccaguuc....................	2	1	Mm2
....aGgcaccagguuauaccaguuc....................	11	1	Mm2
....aagUaccagguuauaccaguuc....................	6	1	Mm2
....aaAcaccagguuauaccaguuc....................	9	1	Mm2
....aaCcaccagguuauaccaguuc....................	4	1	Mm2
....aagcaccagguuaAaccaguuc....................	8	1	Mm2
....aagcaccagguAauaccaguuc....................	3	1	Mm2
....aagcaccUgguuauaccaguuc....................	5	1	Mm2
....Cagcaccagguuauaccaguuc....................	3	1	Mm2
....aagcaccagguuCuaccaguuc....................	2	1	Mm2
....aagcaccCgguuauaccaguuc....................	1	1	Mm2
....aagcacNagguuauaccaguuc....................	2	1	Mm2
....Gagcaccagguuauaccaguuc....................	8	1	Mm2
....aagcaccagguuauaccCguuc....................	134	1	Mm2
....aCgcaccagguuauaccaguuc....................	2	1	Mm2
....aagcaccagUuuauaccaguuc....................	4	1	Mm2
....aagcaccagguuauaccaguuA....................	8	1	Mm2
....aagcaccagguuauGccaguuc....................	4	1	Mm2
....aagcaUcagguuauaccaguuc....................	9	1	Mm2
....aagcaccagAuuauaccaguuc....................	11	1	Mm2
....aagcaccagguuauaccaguuU....................	22	1	Mm2
....aagcaccaggCuauaccaguuc....................	39	1	Mm2
....aagcaccaggGuauaccaguuc....................	50	1	Mm2
....aagcaGcagguuauaccaguuc....................	1	1	Mm2
....aagcGccagguuauaccaguuc....................	4	1	Mm2
....aagcaccagguuauacAaguuc....................	174	1	Mm2
....aagcUccagguuauaccaguuc....................	1	1	Mm2
....aagcaccagguuauaccUguuc....................	112	1	Mm2

```
                                 Star                                Mature
aaggcuuacccuguccuagcaucaguuuugcgaacuggaauaucuugugugcucuuuguauuguaaucaaagcaccagguuauaccaguucgugaucugguccaugcaaua
....................................................................aagcaccagguCauaccaguuc....................	1	1	Mm2
....................................................................aagcaccagguuGuaccaguuc....................	4	1	Mm2
....................................................................aagcacUagguuauaccaguuc....................	13	1	Mm2
....................................................................aagcaccagguuauaccaUuuc....................	5	1	Mm2
....................................................................aagcaccagCuuauaccaguuc....................	1	1	Mm2
....................................................................aagcaccagguuauacGaguuc....................	53	1	Mm2
....................................................................aagcaccaUguuauaccaguuc....................	4	1	Mm2
....................................................................aagcacAagguuauaccaguuc....................	1	1	Mm2
....................................................................aagcaccagguuaGaccaguuc....................	1	1	Mm2
....................................................................aagcaccagguuauaUcaguuc....................	9	1	Mm2
....................................................................aagcaccagguuauaccaCuuc....................	1	1	Mm2
....................................................................aagcaccagguuauaccaguucU...................	1552	1	Mm2
....................................................................aagcaccagguuauaccaguucC...................	67	1	Mm2
....................................................................aagcaccagguuauaccaguucg...................	15	0	Mm2
....................................................................aagcaccagguuauaccaguucUu..................	38	1	Mm2
.....................................................................agcaccagguuauaccaguu.....................	1	0	Mm2
.....................................................................agcacUagguuauaccaguuc....................	1	1	Mm2
.....................................................................aAcaccagguuauaccaguuc....................	1	1	Mm2
.....................................................................agcaccagguuauaccaguuc....................	26	0	Mm2
.....................................................................agcaccagguuauaccaguucU...................	2	1	Mm2
......................................................................gcaccagguuauaccaguu.....................	1	0	Mm2
.......................................................................caccagguuauaccaguuc....................	1	0	Mm2

....................................................................aagcaccagguuauacca........................	19	0	Mm1
....................................................................aagcaccagguuauaccCg.......................	1	1	Mm1
....................................................................aagcaccagguuauaccag.......................	5	0	Mm1
....................................................................aagcaccagguuauaccagu......................	6	0	Mm1
....................................................................aagcaccagguuauaccCguu.....................	1	1	Mm1
....................................................................aagcacUagguuauaccaguu.....................	1	1	Mm1
....................................................................aagcaccagguuauaccUguu.....................	1	1	Mm1
....................................................................aagcaccagguuauaccaguu.....................	255	0	Mm1
....................................................................aagcaccagguuauacUaguu.....................	1	1	Mm1
....................................................................aagcaccaggGuauaccaguu.....................	1	1	Mm1
....................................................................aagcaccagguuauacGaguu.....................	1	1	Mm1
....................................................................aagcaccagguuauacAaguu.....................	2	1	Mm1
....................................................................aagcaccagguuauaccGguu.....................	1	1	Mm1
....................................................................aagcaccUgguuauaccaguuc....................	1	1	Mm1
....................................................................aagcaccagguuauaccGguuc....................	27	1	Mm1
....................................................................aagcaccagguuauaccCguuc....................	27	1	Mm1
....................................................................aagcacNagguuauaccaguuc....................	1	1	Mm1
....................................................................aagcaccagguuUuaccaguuc....................	1	1	Mm1
....................................................................aagcaccagguuauUccaguuc....................	3	1	Mm1
....................................................................aagcaccagguuauaccaguuc....................	6477	0	Mm1
....................................................................aagcaNcagguuauaccaguuc....................	1	1	Mm1
....................................................................aagcaccagguuauaccaUuuc....................	1	1	Mm1
....................................................................aagcaccaggAuauaccaguuc....................	13	1	Mm1
....................................................................aagcaccagguuGuaccaguuc....................	1	1	Mm1
....................................................................aCgcaccagguuauaccaguuc....................	1	1	Mm1
....................................................................aagAaccagguuauaccaguuc....................	1	1	Mm1
....................................................................aagcaccagguuauCccaguuc....................	1	1	Mm1
....................................................................aagcaccaggGuauaccaguuc....................	5	1	Mm1
....................................................................aagcaccagguuauaccaguCc....................	1	1	Mm1
....................................................................aagcaccagAuuauaccaguuc....................	2	1	Mm1
....................................................................aagcaccagguuauacAaguuc....................	24	1	Mm1
....................................................................aagcaccagguuaAaccaguuc....................	4	1	Mm1
....................................................................aagcaccagguuauaccUguuc....................	21	1	Mm1
....................................................................aagcacUagguuauaccaguuc....................	2	1	Mm1
....................................................................Nagcaccagguuauaccaguuc....................	5	1	Mm1
....................................................................aagcaccagguuauacGaguuc....................	11	1	Mm1
....................................................................aagcaccaggCuauaccaguuc....................	6	1	Mm1
....................................................................aUgcaccagguuauaccaguuc....................	1	1	Mm1
....................................................................aagcaccagguuauGccaguuc....................	1	1	Mm1
....................................................................Uagcaccagguuauaccaguuc....................	4	1	Mm1
....................................................................aagcaccagguCauaccaguuc....................	2	1	Mm1
....................................................................aagcGccagguuauaccaguuc....................	1	1	Mm1
....................................................................aaAcaccagguuauaccaguuc....................	3	1	Mm1
....................................................................aGgcaccagguuauaccaguuc....................	1	1	Mm1
....................................................................aagcaccagguuauaccaguuU....................	3	1	Mm1
....................................................................aaUcaccagguuauaccaguuc....................	2	1	Mm1
....................................................................aagcaccaAguuauaccaguuc....................	1	1	Mm1
```

```
                                Star                               Mature
aaggcuuacccuguccuagcaucaguuuugcgaacuggaauaucuugugugcucuuuguauuguaaucaaagcaccagguuauaccaguucgugaucugguccaugcaaua
.....................................................................aagcaccagguuauacUaguuc....................    14    1    Mm1
.....................................................................aagcaccagguuauaccaguucU...................    249   1    Mm1
.....................................................................aagcaccagguuauaccaguucC...................    36    1    Mm1
.....................................................................aagcaccagguuauaccaguucg...................    2     0    Mm1
......................................................................agcaccagguuauaccaguuc....................    1     0    Mm1
......................................................................agcaccagguuauaccaguucU...................    3     1    Mm1
```
